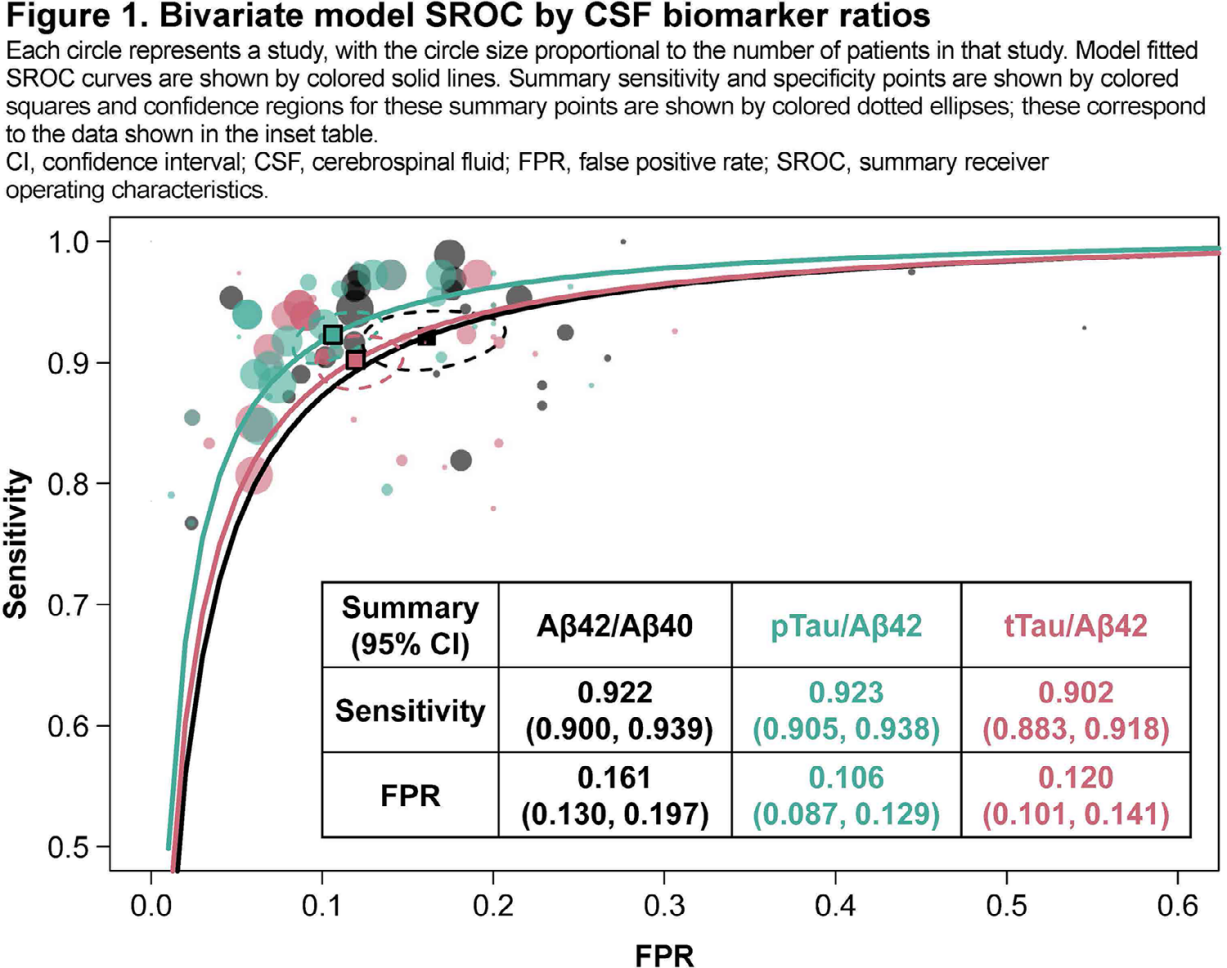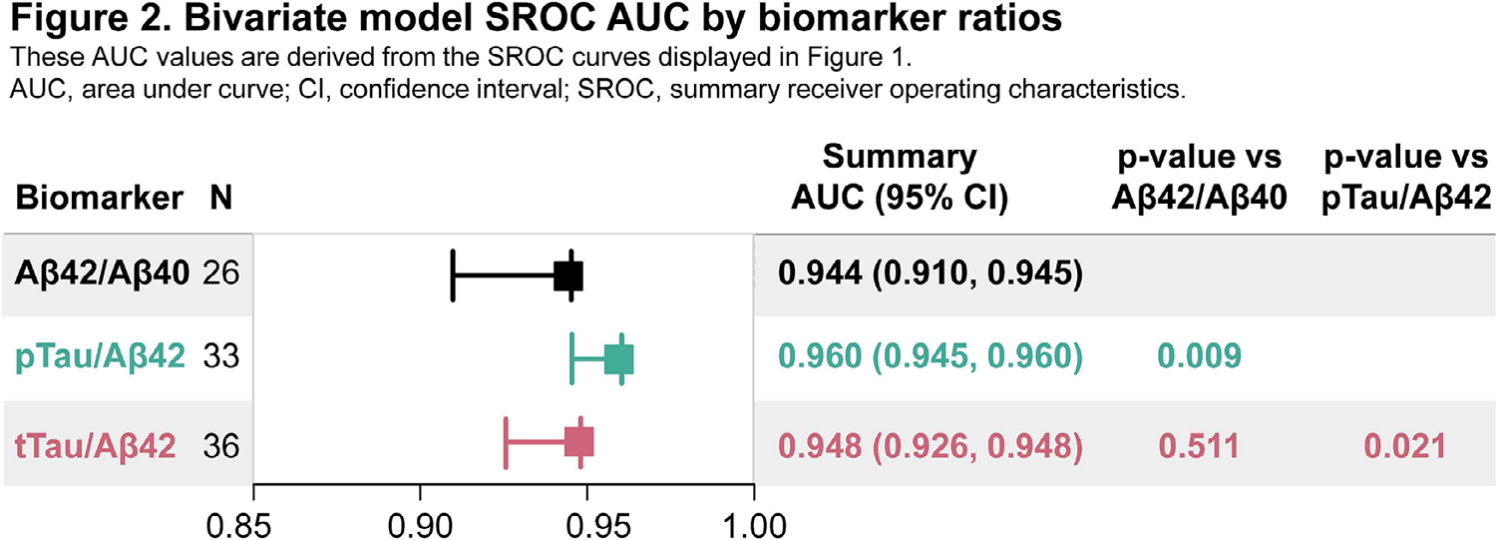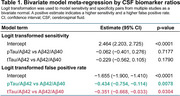# Accuracy of cerebrospinal fluid biomarker ratios to determine amyloid positron‐emission tomography status: a diagnostic test accuracy meta‐analysis

**DOI:** 10.1002/alz70856_100941

**Published:** 2025-12-24

**Authors:** Pablo Martinez‐Lage, Eino Solje, Julian G. Martins, Sraboni Sarkar

**Affiliations:** ^1^ Center for Research and Memory Clinic, CITA‐Alzheimer Foundation, San Sebastián, Gipuzkoa, Spain; ^2^ Institute of Clinical Medicine – Neurology, University of Eastern Finland, Kuopio, Northern Savo, Finland; ^3^ Neuro Center – Neurology, Kuopio University Hospital, Kuopio, Northern Savo, Finland; ^4^ inScience Communications, Springer Nature, Paris, Île‐de‐France, France; ^5^ Global Medical Affairs, Roche Diagnostics International Ltd, Rotkreuz, Zug, Switzerland

## Abstract

**Background:**

Amyloid positron‐emission tomography (PET) is considered a gold standard for confirmation of the diagnosis of Alzheimer's disease (AD). However, its use is limited by cost and scanner availability. Cerebrospinal fluid (CSF) biomarker ratios (Aβ42/Aβ40, pTau/Aβ42, and tTau/Aβ42) are lower‐cost diagnostic alternatives that show high concordance with amyloid PET. Even though Aβ42/Aβ40 is the most studied ratio for AD diagnosis, it is not known whether pTau/Aβ42 or tTau/Aβ42 ratios show equivalent, higher, or lower concordance with amyloid PET. To evaluate this, we conducted a diagnostic test accuracy (DTA) meta‐analysis.

**Methods:**

Medline and Embase were searched for articles from January 2008 to October 2024 that reported concordance of amyloid PET with at least one CSF biomarker ratio. Extracted data were analyzed according to methods for DTA meta‐analysis outlined by Cochrane collaboration. Three bivariate model outcomes evaluating CSF biomarker ratio concordance with amyloid PET were performed: summary receiver operating characteristics (SROC) curves, SROC area under the curve (AUC) values, and meta‐regression. Statistical analyses were performed using the bivariate model of Reitsma with the R package mada v0.5.11.

**Results:**

We included 31 studies comprising 6,238 patients. Most studies were prospective and observational in design (64.5%) and were primarily conducted in Europe (45.2%) or North America (25.8%). In these 31 studies, 11.1–100% of patients had cognitive impairment, and 25.3–87.9% were amyloid PET positive. SROC curves demonstrated sensitivity values of 0.922, 0.923, and 0.902 for Aβ42/Aβ40, pTau/Aβ42, and tTau/Aβ42 ratios, respectively; false positive rates were 0.161, 0.106, and 0.120, respectively (Figure 1). SROC AUC values for Aβ42/Aβ40, pTau/Aβ42, and tTau/Aβ42 ratios were 0.944, 0.960 (*p* = 0.009 vs Aβ42/Aβ40), and 0.948 (*p* = 0.021 vs pTau/Aβ42), respectively (Figure 2). Meta‐regression demonstrated significantly lower false positive rates versus Aβ42/Aβ40 for pTau/Aβ42 (*p* = 0.0078) and tTau/Aβ42 (*p* = 0.0304; Table 1).

**Conclusions:**

All three CSF biomarker ratios (Aβ42/Aβ40, pTau/Aβ42, and tTau/Aβ42) analyzed were highly concordant with amyloid PET. However, the pTau/Aβ42 ratio consistently showed higher concordance with amyloid PET positivity than the Aβ42/Aβ40 or tTau/Aβ42 ratio across multiple analysis methods, suggesting that pTau/Aβ42 may be the preferred CSF biomarker ratio for confirmation of the diagnosis of AD.